# A Ribosomal Protein Homolog Governs Gene Expression and Virulence in a Bacterial Pathogen

**DOI:** 10.1128/jb.00268-22

**Published:** 2022-09-19

**Authors:** Hannah S. Trautmann, Kathryn M. Ramsey

**Affiliations:** a Department of Cell and Molecular Biology, University of Rhode Island, Kingston, Rhode Island, USA; b Department of Biomedical and Pharmaceutical Sciences, University of Rhode Island, Kingston, Rhode Island, USA; University of Notre Dame

**Keywords:** *Francisella*, gene regulation, posttranscriptional control mechanisms, ribosomal proteins, virulence regulation

## Abstract

The molecular machine necessary for protein synthesis, the ribosome, is generally considered constitutively functioning and lacking any inherent regulatory capacity. Yet ribosomes are commonly heterogeneous in composition and the impact of ribosome heterogeneity on translation is not well understood. Here, we determined that changes in ribosome protein composition govern gene expression in the intracellular bacterial pathogen Francisella tularensis. F. tularensis encodes three distinct homologs for bS21, a ribosomal protein involved in translation initiation, and analysis of purified F. tularensis ribosomes revealed they are heterogeneous with respect to bS21. The loss of one homolog, bS21-2, resulted in significant changes to the cellular proteome unlinked to changes in the transcriptome. Among the reduced proteins were components of the type VI secretion system (T6SS), an essential virulence factor encoded by the Francisella Pathogenicity Island. Furthermore, loss of bS21-2 led to an intramacrophage growth defect. Although multiple bS21 homologs complemented the loss of bS21-2 with respect to T6SS protein abundance, bS21-2 was uniquely necessary for robust intramacrophage growth, suggesting bS21-2 modulates additional virulence gene(s) distinct from the T6SS. Our results indicate that ribosome composition in F. tularensis, either directly or indirectly, posttranscriptionally modulates gene expression and virulence. Our findings are consistent with a model in which bS21 homologs function as posttranscriptional regulators, allowing preferential translation of specific subsets of mRNAs, likely at the stage of translation initiation. This work also raises the possibility that bS21 in other organisms may function similarly and that ribosome heterogeneity may permit many bacteria to posttranscriptionally regulate gene expression.

**IMPORTANCE** While bacterial ribosomes are commonly heterogeneous in composition (e.g., incorporating different homologs for a ribosomal protein), how heterogeneity impacts translation is unclear. We found that the intracellular human pathogen Francisella tularensis has heterogeneous ribosomes, incorporating one of three homologs for ribosomal protein bS21. Furthermore, one bS21 homolog posttranscriptionally governs the expression of the F. tularensis type VI secretion system, an essential virulence factor. This bS21 homolog is also uniquely important for robust intracellular growth. Our data support a model in which bS21 heterogeneity leads to modulation of translation, providing another source of posttranscriptional gene regulation. Regulation of translation by bS21, or other sources of ribosomal heterogeneity, may be a conserved mechanism to control gene expression across the bacterial phylogeny.

## INTRODUCTION

Regulation of translation provides bacteria with a rapid way to modify gene expression. While many distinct mechanisms permit this fine-tuning (1, 2) the impact of ribosome composition on gene expression remains poorly understood. In bacteria, ribosomes are diverse and commonly heterogeneous with respect to ribosomal protein (r-protein) content, posttranslational modifications, rRNA content, or posttranscriptional modifications (reviewed in reference (3)). The functional consequences of ribosome heterogeneity are unclear but may include the formation of “specialized ribosomes,” or ribosomes with altered activity due to their distinct composition (4). Although specialized ribosomes are not well described in bacteria, exciting recent studies have connected altered rRNA content of ribosomes and gene regulation (5, 6) and, in Mycobacterium smegmatis, ribosomes containing alternate r-protein homologs translate some genes with differential efficiency (7).

Francisella tularensis is a Gram-negative, facultative intracellular bacterium that causes the potentially fatal human disease tularemia (8). After internalization into host cells, F. tularensis must escape from the Francisella-containing phagosome to replicate inside the cytosol. This escape process requires a type VI secretion system (T6SS), which modifies the host cell by delivery of effector proteins (9–12). Production of this T6SS is coordinately regulated by the transcription factors MglA, SspA, and PigR, as well as the signaling molecule ppGpp (13–20). Regulation of the T6SS is arguably the most well-understood virulence regulatory network in F. tularensis. However, much remains to be learned about the regulation of other virulence factors.

Despite its relatively small genome (<2 Mbp), F. tularensis encodes three distinct *rpsU* genes (*rpsU1*, *rpsU2*, and *rpsU3*), which encode homologs of the small ribosomal subunit protein bS21 (bS21-1, bS21-2, and bS21-3, respectively). This is the only apparent source of ribosome heterogeneity in F. tularensis, as the three rRNA operon sequences are identical and no other r-proteins are encoded by multiple homologs. In Escherichia coli, bS21 is involved in translation initiation (21, 22) and, consistent with this activity, is found on the ribosome close to the anti-Shine-Dalgarno sequence near the mRNA exit channel (23, 24). Furthermore, bS21 is one of the last r-proteins to assemble into the ribosome, is considered “loosely associated,” and is easily exchanged among assembled ribosomes (25, 26).

Using mass spectrometry and immunoblot analyses, we showed that ribosomes in F. tularensis are heterogeneous with respect to bS21 content and can incorporate any of the three bS21 homologs into actively translating ribosomes. Using quantitative whole-cell proteomics, quantitative immunoblots, and transcriptomic analyses, we demonstrated that loss of a particular bS21 homolog, bS21-2, leads to changes in abundance for a subset of proteins that can not be explained by changes in transcript abundance. Among the impacted proteins are multiple virulence factors, including those that comprise the T6SS. Finally, using intramacrophage growth assays, we provide evidence that bS21-2, and not the other bS21 homologs, promotes intramacrophage growth. Our findings reveal that a specific r-protein homolog in F. tularensis, bS21-2, governs gene expression at the level of protein abundance and positively impacts virulence.

## RESULTS

### *Francisella* species encode three bS21 homologs.

The genomes of multiple *Francisella* species contain three distinct genes encoding bS21 (*rpsU1*, *rpsU2*, and *rpsU3*), raising the possibility that cells contain ribosomes that are heterogeneous with respect to bS21 content. The gene encoding one homolog in F. tularensis, *rpsU2* (encoding bS21-2), is syntenic with the single bS21-encoding gene in Escherichia coli (Fig. S1 in Supplemental File 1). In E. coli, *rpsU* is the first in an operon referred to as the macromolecular synthesis operon, encoding key proteins for initiation of translation (bS21), DNA replication (DNA primase), and transcription (RNA polymerase σ^70^) (27). The corresponding operon in *Francisella* species, including F. tularensis, also contains *yqeY*, which may encode a protein necessary for correct tRNA aminoacylation (28). Another bS21 homolog, bS21-1, is encoded by *rpsU1* in an apparent operon downstream of the gene for cold shock protein CspC. There are no annotated genes in the same transcriptional context as *rpsU3*, the gene encoding the third homolog, bS21-3. The bS21 homologs in F. tularensis are distinct but similar, with amino acid identities ranging from 48 to 72%, and are similar to E. coli bS21 (51 to 60% identical, with bS21-2 having the highest identity; Fig. S2 in Supplemental File 1).

### F. tularensis ribosomes are heterogeneous.

**FIG 1:**
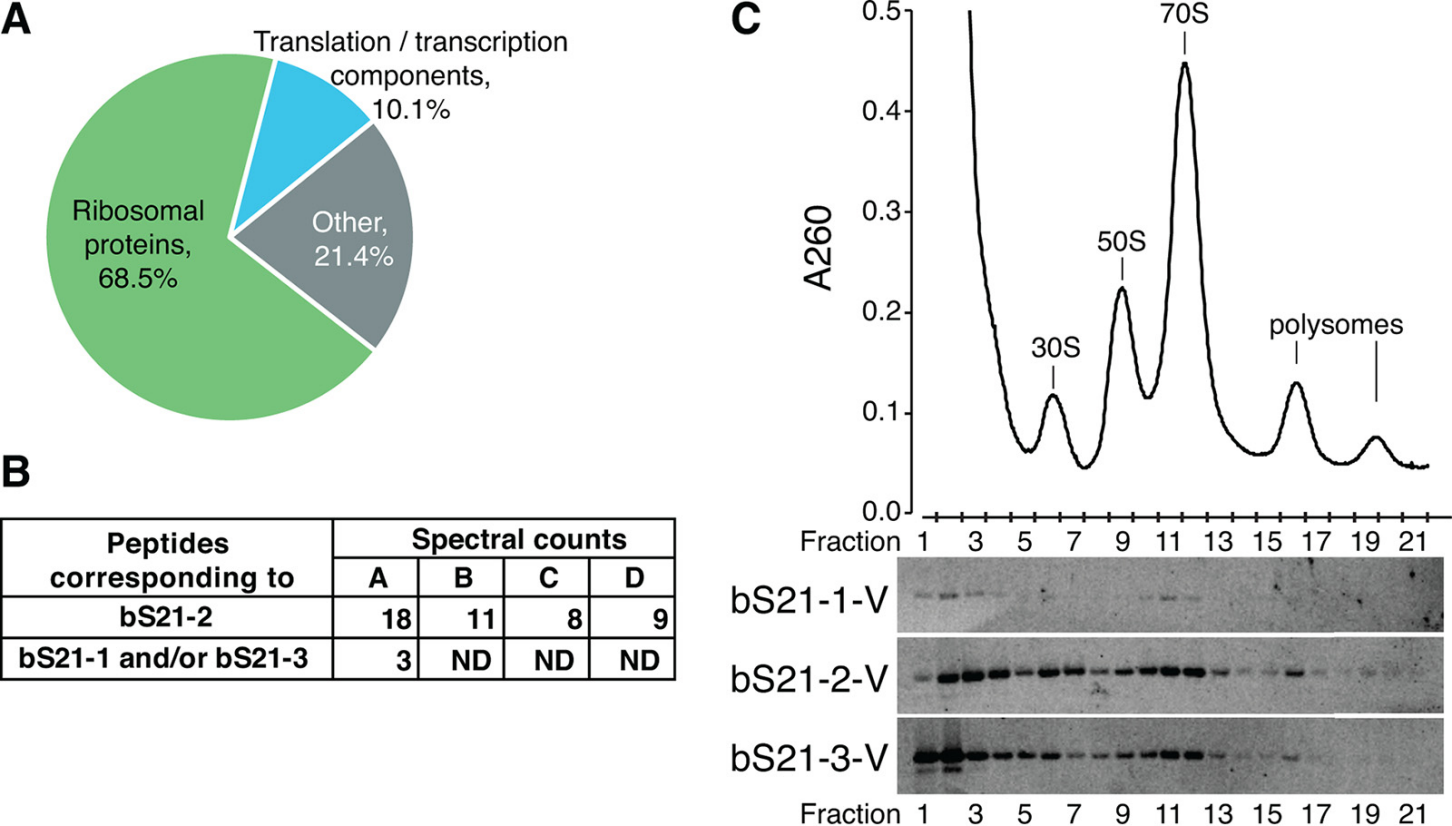
F. tularensis ribosomes are heterogeneous with respect to bS21. (A) A chart demonstrating the purity of wild-type ribosomes. Categories represent the classification of proteins identified by mass spectrometry of ribosomes purified from wild-type F. tularensis LVS cells. Numbers represent the percentage of spectral counts corresponding to proteins in each category, combined from quadruplicate samples. (B) Wild-type F. tularensis LVS ribosomes contain more than one bS21 homolog. A table detailing the number of spectral counts corresponding to bS21 homologs identified from individual ribosome purifications (A to D) from wild-type cells. Spectral counts corresponding to bS21-1 and/or bS21-3 could not be unambiguously assigned due to the complete sequence identity of detected peptides. ND: not detected. (C) Each bS21 homolog could be incorporated into ribosomes. Top, sucrose gradient sedimentation profile from actively translating wild-type cells containing an empty vector. Nucleic acid content was monitored by the absorbance at 260 nm (A_260_) (*y*-axis). Peaks corresponding to the 30S, 50S, 70S, and polysomes are indicated. Fractions collected are indicated on the *x*-axis. Bottom, immunoblot analysis of fractions from sucrose gradient sedimentation performed on actively translating cells ectopically expressing indicated bS21 homolog with VSV-G epitope tag. Wells correspond to fractions 1 to 21 from the profile above.

The presence of three distinct genes encoding bS21 raises the potential for F. tularensis ribosomes to be heterogenous with respect to bS21. To investigate this possibility, we used sucrose cushion centrifugation to isolate ribosomes from F. tularensis LVS grown *in vitro* in quadruplicate and analyzed their protein composition using liquid chromatography-tandem mass spectrometry (LC-MS/MS). Approximately 80% of the spectral counts corresponded to ribosomal proteins or proteins associated with transcription and translation complexes (e.g., RNA polymerase, translation release factors, SRP), indicating F. tularensis ribosomes purified in this manner are highly pure (Fig. 1A, Table S1 in Supplemental File 2). Despite the small size of bS21 (approximately 8 kDa), we identified multiple peptides corresponding to bS21-2 in all samples. In one sample, peptides shared between bS21-1 and bS21-3 were detected (Fig. 1B). This suggests that bS21-2 is the most abundant homolog in wild-type cells, consistent with its production from operon encoding proteins essential for transcription and DNA replication. It also suggests that either bS21-1, bS21-3, or both, are incorporated into ribosomes in LVS. However, it did not allow us to determine the next-most abundant homolog (bS21-1 or bS21-3) or confirm the incorporation of both other homologs. Regardless, these results demonstrate that multiple bS21 homologs are incorporated into wild-type F. tularensis ribosomes and that ribosomes in F. tularensis are heterogenous, containing different bS21 homologs.

We next wanted to determine if each bS21 homolog could be found in actively translating ribosomes. To track each bS21, we modified each homolog to encode a C-terminal vesicular stomatitis virus glycoprotein (VSV-G) tag and individually ectopically expressed them, using the same promoter, from a plasmid in wild-type cells. Lysate fractions of these cells were analyzed by immunoblotting after sucrose gradient sedimentation (Fig. 1C; Fig. S3 in Supplemental File 1). When ectopically expressed (rather than produced from its native locus), bS21-1 was the least abundant homolog while bS21-3 was produced at the highest level. Each homolog was found in fractions corresponding to the 30S, 70S, and polysomes. Although bS21 is thought to function primarily in translation initiation, our findings indicate that each bS21 homolog associates with the ribosome throughout the translation cycle.

### Loss of bS21-2 leads to changes in protein, but not transcript, abundance.

**FIG 2:**
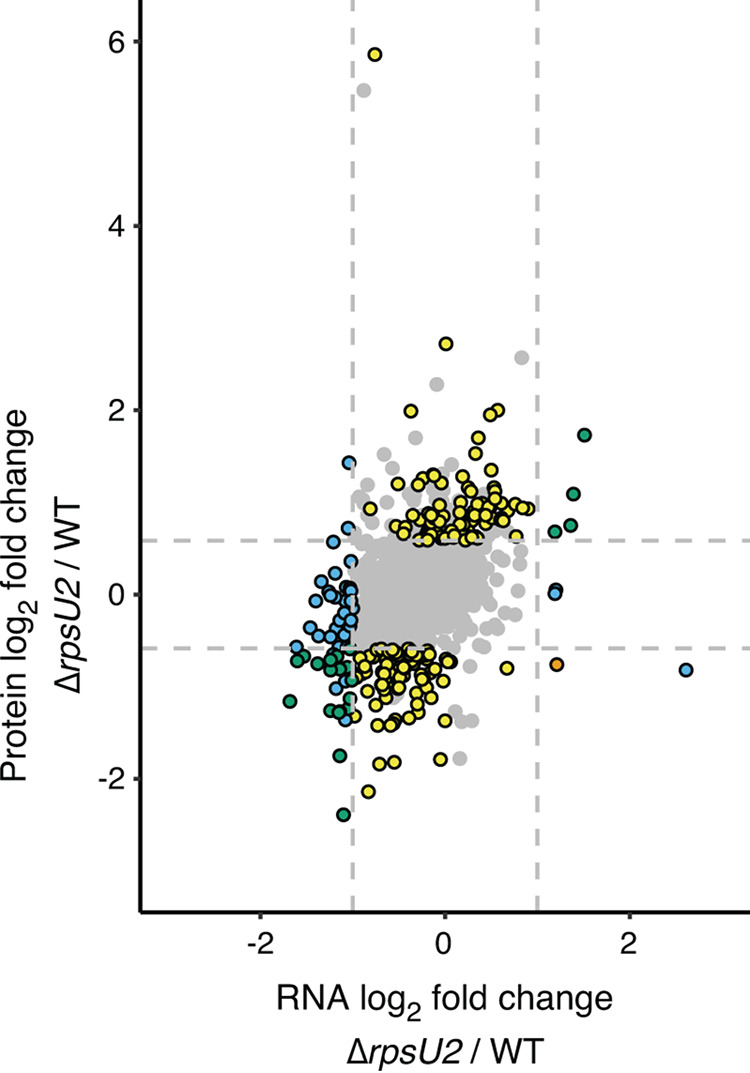
Loss of bS21-2 leads to changes in protein abundance that can not be explained by changes in transcript abundance. Cells with (WT, wild-type) and without bS21-2 (Δ*rpsU2*) were analyzed using RNA-Seq (*x*-axis) and DIA whole-cell mass spectrometry (*y*-axis). Genes are represented by dots. Most genes with changes in protein (161 yellow dots) did not have corresponding changes in transcript abundance. One gene (orange dot) had discordant changes in transcript and protein abundance. Green dots (23) represent genes with concordant changes in transcript and protein abundance. Blue dots (60) indicate genes with altered transcript abundance only. Horizontal dashed lines indicate a ±1.5-fold cutoff for differential protein abundance; vertical dashes indicate a ±2-fold cutoff for differential transcript abundance. Colored dots with black outlines represent genes with significant changes in protein (±1.5-fold change, adjusted *P* < 0.05) and/or transcript (±2-fold change, adjusted *P* < 0.05) abundance as indicated above, while gray dots without outlines represent genes with changes that did not meet the statistical thresholds. Three gray dots are located outside the bounds of the axes as represented.

Because the ribosomal protein bS21 is involved in translation initiation, we hypothesized that loss of a bS21 homolog may impact translation and result in changes in abundance in a subset of proteins. To test this hypothesis, we individually deleted each of the three genes encoding bS21 homologs. This led us to determine that no single bS21 homolog is essential for cell growth. We subsequently grew wild-type cells and cells lacking single bS21 homologs to the mid-log phase *in vitro* and used data-independent acquisition (DIA) mass spectrometry analysis (29) to compare relative protein abundance in cell lysates. Using this method, 68% of the total proteins predicted to be encoded by F. tularensis LVS were identified and analyzed (1194 of 1754). Compared to wild-type, we did not detect any significant changes in protein abundance in cells lacking either of the two lower-abundance bS21 homologs, bS21-1 and bS21-3 (>1.5-fold altered with an adjusted *P* < 0.05, excluding bS21). In contrast, cells lacking the most abundant homolog, bS21-2 (Δ*rpsU2*), had significant proteomic differences compared to wild-type cells. Specifically, we found 185 unique proteins (~16% of detected proteins) had altered abundance in cells without bS21-2 compared to wild-type cells (Fig. 2, data on the *y*-axis; Table S2 in Supplemental File 3).

To determine if these changes in protein abundance could be explained by corresponding changes in transcription, we performed transcriptomic analyses on wild-type cells, cells lacking bS21-2 (Δ*rpsU2*), and cells lacking the native bS21-2 but ectopically expressing bS21-2-V from a plasmid. Comparing cells with and without native bS21-2, we identified 105 differentially expressed genes (>2-fold altered with an adjusted *P* < 0.05, excluding *rpsU*; Fig. 2, data on the *x*-axis, Table S3 in Supplemental File 4). All these changes were complemented by ectopic expression of bS21-2-V on a plasmid.

Our analysis revealed that in cells lacking bS21-2, the largest change in transcript abundance was a 6-fold increase in *yqeY*, the gene directly downstream from *rpsU2*, which encodes bS21-2. This increase in transcript abundance was complemented by the ectopic expression of bS21-2-V, suggesting that bS21-2 functions as a negative regulator of its own operon. Translational feedback regulation is well-established for multiple ribosomal proteins (30, 31), but, to the best of our knowledge, this is the first report of translational regulation of ribosomal proteins in F. tularensis and that bS21 governed its own production.

A comparison of our proteomic and transcriptomic analyses revealed that the changes in protein abundance were not generally due to changes in transcript abundance. Of the 185 differentially abundant proteins in cells lacking bS21-2, only about 12% (23) could be explained by altered transcription (Fig. 2, yellow dots), while about 88% (162; Fig. 2, blue dots and orange dot) had changes in protein abundance without a corresponding change in transcript abundance. These discrepancies between transcript abundance and protein abundance support a model in which bS21-2 controls the expression, either directly or indirectly, of some genes at the level of translation.

### bS21-2 governs the abundance of type VI secretion system proteins, which are essential for virulence.

**FIG 3:**
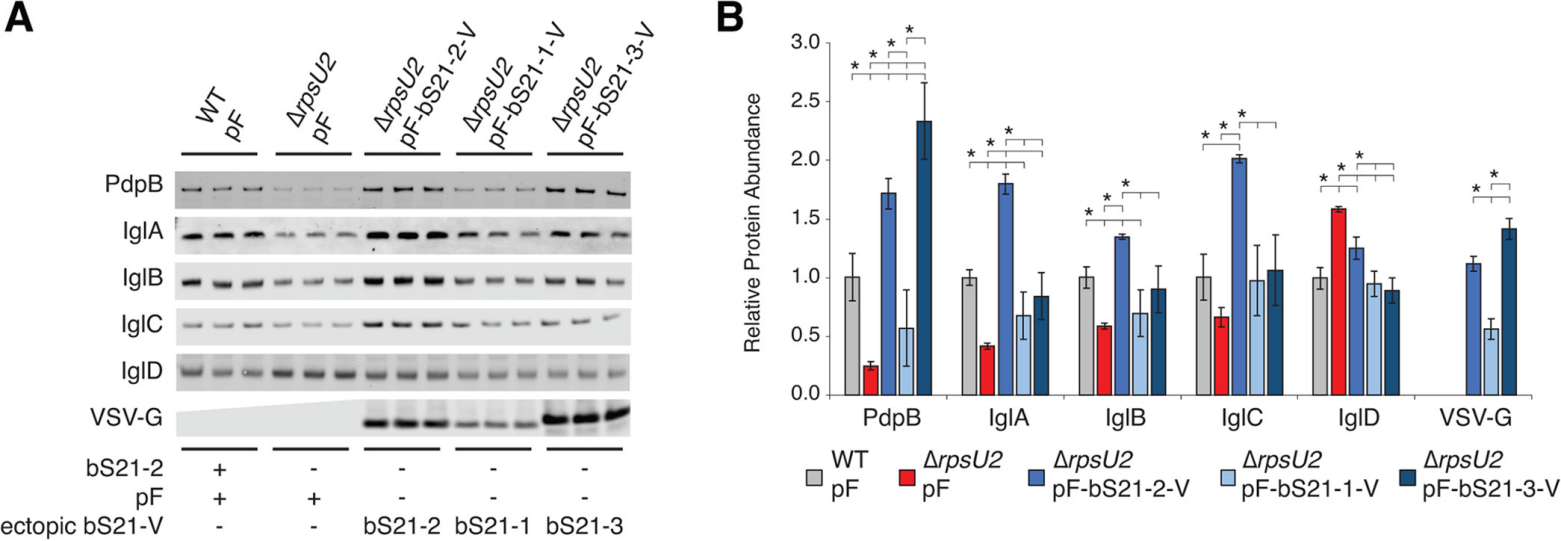
bS21-2 impacts T6SS protein abundance. (A) Immunoblot analysis of indicated T6SS protein abundance. Cells either contained (WT, wild-type) or lacked (Δ*rpsU2*) bS21-2 and either an empty vector control (pF) or a vector ectopically expressing VSV-G-tagged bS21 homologs (pF-bS21-1-V, pF-bS21-2-V, or pF-bS21-3-V). Immunoblot against VSV-G was included to demonstrate the production of VSV-G-tagged bS21 homologs. (B) Quantification of immunoblots from (A). Band intensities for each protein were normalized to total protein per well on the membrane. Error bars represent 1 SD. Experiments were repeated at least twice and data from a representative experiment are shown. The lines above bars indicate statistical comparison among groups by *t* test. Asterisk indicates the group to which all other groups are compared if the horizontal line connects to the line above group; *, *P* < 0.05 using Benjamini-Hochberg correction.

Among the proteins with altered abundance in cells lacking bS21-2, we identified 12 out of 16 proteins encoded on the Francisella Pathogenicity Island (FPI). The FPI encodes a unique type VI secretion system (T6SS) that is essential for intramacrophage growth and virulence of F. tularensis (32–34). Using quantitative immunoblotting and antibodies specific to a subset of F. tularensis T6SS proteins, we validated that cells lacking bS21-2 have differences in those T6SS proteins (Fig. 3). Consistent with the mass spectrometry results, we found reductions in virtually all probed T6SS proteins, including an ~4-fold reduction in PdpB, the TssM/IcmF homolog. Using this approach, we also found an ~2.4-fold reduction in IglA and ~1.7-fold reduction in IglB, T6SS proteins that were just below the cutoff for statistical significance in our mass spectrometry analysis. Because we identified this differential abundance using a more sensitive method of comparison, it raises the possibility that all FPI-encoded proteins may be differentially abundant in cells lacking bS21-2 compared to wild-type cells, but we do not have antibodies specific to the remaining proteins (i.e., PdpE and VgrG) to test this hypothesis. Also consistent with our mass spectrometry findings, IglD (the homolog of TssK) was the only T6SS protein with increased, rather than decreased, protein abundance (Fig. 3). Each of these changes in protein abundance could be complemented by ectopic expression of bS21-2-V, driven by the *groES* promoter on a plasmid (Fig. 3).

These changes in protein abundance likely reflect positive regulation of most, but not all, T6SS proteins by bS21-2 at the level of translation, either directly or indirectly. Our findings are inconsistent with bS21 positively regulating transcription. It is well-established that transcription of FPI operons is coordinately controlled, and our RNA-Seq analysis revealed that cells lacking bS21-2 did not have FPI-wide transcript reductions (13–16, 20, 35, Table S3 in Supplemental File 4). In a complementary approach, we compared the transcript abundance for specific FPI genes using quantitative RT-PCR and included cells lacking PigR, a transcription factor critical for positive transcriptional regulation of FPI genes (14–16, 20, 35, Fig. S4 in Supplemental File 1). We confirmed that cells lacking PigR have major decreases in FPI transcript abundance, but cells lacking bS21-2 did not have compelling (2-fold or greater) changes in FPI transcript abundance or transcript abundance of the positive regulator PigR, consistent with the RNA-Seq results. We considered the possibility that loss of bS21-2 could indirectly impact T6SS protein abundance by altering protein stability, but the half-life of one of the most differentially abundant proteins, PdpB, was unchanged in cells with and without bS21-2 (longer than 120 min, Fig. S5 in Supplemental File 1). Our results are consistent with bS21-2 controlling the expression of T6SS proteins at the level of translation.

### Other bS21 homologs impact the abundance of type VI secretion system proteins.

Our findings indicate that bS21-2 is the most abundant bS21 homolog in wild-type cells. However, it is not clear if most ribosomes in cells lacking bS21-2 incorporate another bS21 homolog or no bS21 at all. This leads to the question: do all bS21 homologs affect T6SS protein translation or does bS21-2 specifically modulate the translation of T6SS proteins? To answer this question, we ectopically expressed either bS21-1-V or bS21-3-V in cells lacking bS21-2, similarly to the ectopic expression of bS21-2-V. We subsequently used quantitative immunoblot analyses to assess the abundance of each ectopically expressed bS21 homolog and a subset of T6SS proteins (Fig. 3). While this strategy resulted in comparable amounts of bS21-2 and bS21-3, ectopic expression resulted in approximately 2-fold less bS21-1 than the other homologs, consistent with its lower expression in wild-type cells (Fig. 1 and 3). With respect to T6SS protein abundance, ectopic expression of bS21-3 restored all probed proteins to wild-type levels, complementing the loss of bS21-2 (Fig. 3). However, bS21-1 did not appear to complement T6SS protein production completely (Fig. 3). This may be due to reduced levels of bS21-1, lack of specific ability to regulate T6SS proteins, or a combination of the two factors. Notably, loss of bS21-2 resulted in a growth defect (Table S4 in Supplemental File 5) that could be complemented by ectopic expression of bS21-2 or bS21-1, but not bS21-3. The fact that cells lacking bS21-2 with ectopic expression of bS21-3 had wild-type levels of T6SS proteins and yet still had a growth defect revealed that changes in T6SS proteins are not due simply to changes in growth rate. Our findings allow us to conclude that incorporation of either bS21-2 or bS21-3 – and to a lesser extent, bS21-1 – into ribosomes modulates the production of T6SS proteins.

### bS21-2 is important for intramacrophage growth.

**FIG 4:**
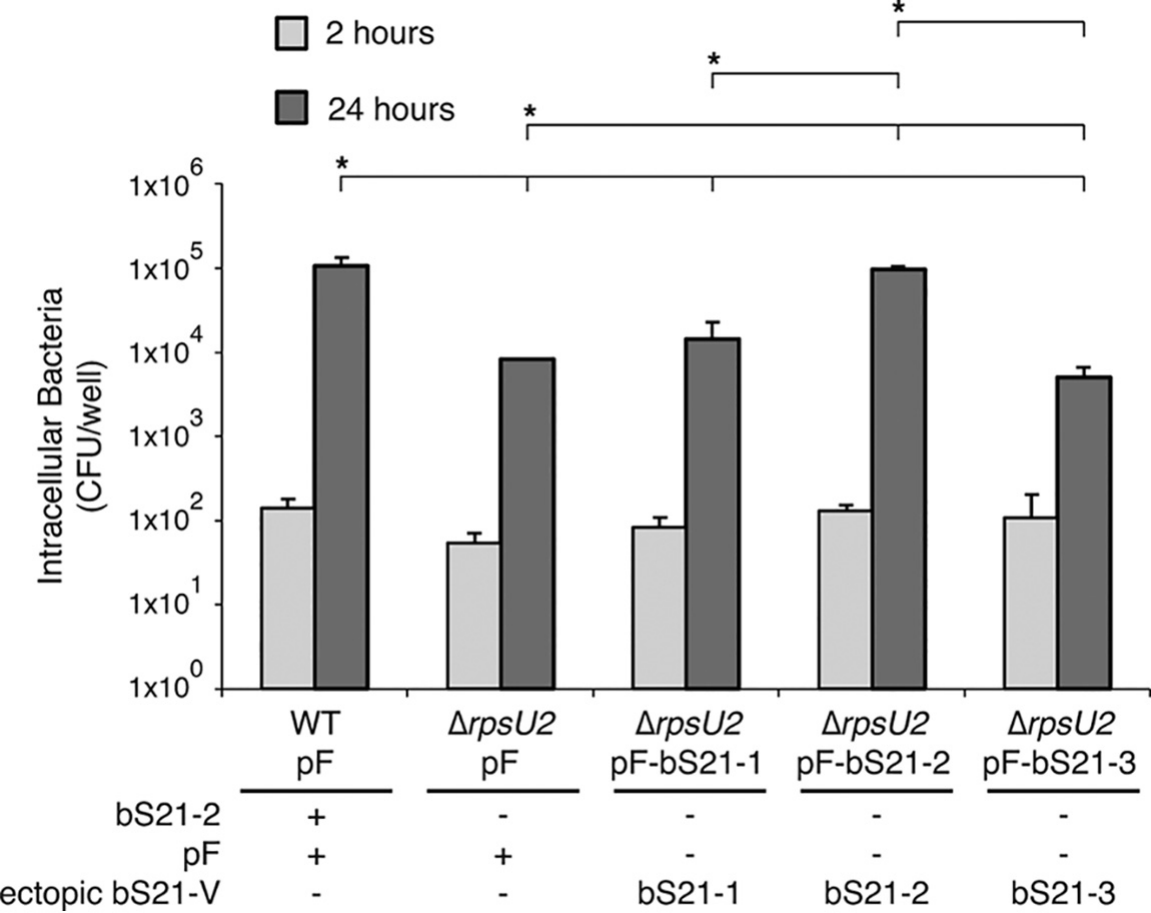
Cells without bS21-2 have an intramacrophage growth defect, which can be complemented by the ectopic expression of bS21-2. Growth and survival of F. tularensis LVS cells within J774A.1 cells. Murine macrophage-like J774A.1 cells were infected with indicated bacterial cells at a multiplicity of infection of 5 to 10. J774A.1 cells were lysed, and bacteria were plated for enumeration (CFU) at 2 h and 24 h postinfection. Error bars represent 1 SD. Experiments were repeated at least twice and data from a representative experiment are shown. The lines above bars indicate statistical comparison among groups by *t* test. An asterisk indicates the group to which all other groups were compared if the horizontal line connects to the line above the group; *, *P* < 0.05 using Benjamini-Hochberg correction.

A functional T6SS is essential for F. tularensis intramacrophage replication and is a strict requirement for virulence (32–34). The observed differences in FPI protein abundance led us to hypothesize that T6SS function may be compromised in cells lacking bS21-2 and these cells may be attenuated for intramacrophage growth. We tested the ability of cells lacking bS21-2 (Δ*rpsU2*) to survive in murine macrophage-like J774A.1 cells. This revealed a significant defect in the ability of bS21-2 mutant cells to replicate in macrophage. We recovered 10-fold fewer bS21-2 mutant bacteria after 24 h compared to wild-type (Fig. 4). The intramacrophage growth defect of cells lacking bS21-2 could be restored by ectopic expression of bS21-2 from a plasmid (Fig. 4). This contrasted with the ectopic expression of bS21-1 and bS21-3, neither of which restored the intramacrophage growth of cells lacking bS21-2 (Fig. 4). These results indicate that bS21-2 is specifically required for intramacrophage survival, even though the ectopic expression of bS21-1 restored *in vitro* growth rates and ectopic expression of bS21-3 restored T6SS protein production *in vitro* (Fig. 3; Table S4 in Supplemental File 5).

In summary, only the presence of bS21-2, not bS21-1 or bS21-3, could restore the intramacrophage growth defect of cells without bS21-2. This reveales that bS21-2 is critical for F. tularensis virulence and fits a model in which bS21-2 specifically regulates one or more genes necessary for intramacrophage growth in addition to T6SS genes, a topic still under investigation.

## DISCUSSION

The findings described here reveal that ribosome composition in F. tularensis is heterogeneous with respect to the small ribosomal protein bS21 and that this heterogeneity impacts gene expression at the level of translation. By studying cells that contain ribosomes either with or without one of the three bS21 homologs, we identified that bS21-2 governs the abundance of most T6SS proteins. Additionally, cells lacking bS21-2 are defective for intramacrophage growth. Because this intramacrophage growth defect could only be complemented by bS21-2, even though bS21-3 and (to a lesser extent) bS21-1 could restore T6SS protein abundance, this intramacropahge growth defect is likely independent of the impact bS21-2 has on the T6SS. This allows us to conclude that bS21-2 is important for intramacrophage growth of F. tularensis, potentially by regulating the translation of one or more proteins (in addition to the T6SS) necessary for virulence.

To examine the impact of bS21 homologs on gene expression, we used tools that assess the steady-state abundance of protein and transcripts: mass spectrometry and RNA-Seq, respectively. Our analyses revealed that compared to wild-type cells, cells lacking bS21-2 have changes in protein abundance that could not be explained by steady-state changes in corresponding mRNAs. Yet for some proteins with altered abundance in cells lacking bS21-2, there were corresponding modest differences in transcript abundance that did not reach statistical significance (Fig. 2, yellow dots). Loss of bS21-2 may lead to modest transcript abundance changes that result in more significant changes in protein abundance. But given the role of bS21 in translation, and specifically translation initiation, we propose a model in which bS21-2 impacts gene expression by modulating translation initiation for specific mRNAs. Because an mRNA can be stabilized by translation, increased translation can increase stability and, conversely, less translation can lead to faster degradation (reviewed in reference (36)). This effect may impact the abundance of many transcripts in cells lacking bS21-2 and may explain the observed weak correlation between some protein and transcript abundances. Consequently, additional work will be required to validate our model.

Our approach to studying bS21 homologs in F. tularensis has thus far focused on the homolog bS21-2, whose loss led to phenotypic changes. Our data suggest that bS21-2 is the most abundant homolog under the conditions studied. We hypothesize that cells without bS21-1 or bS21-3 did not exhibit distinct phenotypes under the conditions of our experiments due to their relatively low abundance. Both of these homologs may also influence gene expression under conditions when they are more abundant, but these conditions are not yet identified. Additionally, in our study of cells without bS21-2, it is not clear if the majority of ribosomes lack bS21 entirely or instead incorporate bS21-1 or bS21-3. Our findings only extend to heterogeneity with respect to the presence or absence of bS21-2.

A comparison of *rpsU* genes across the bacterial phylogeny reveals that many clades and species do not encode bS21, suggesting that it is not essential for translation (37, 38). However, targeted deletion of the single *rpsU* gene in E. coli has not been successful, suggesting bS21 is essential in E. coli (39–41). We reported in previous work that the F. tularensis homolog syntenic with E. coli *rpsU*, *rpsU2*, is essential *in vitro* using transposon-insertion sequencing (Tn-Seq) (42). Yet using a targeted allelic exchange approach, we have been able to successfully delete each *rpsU* homolog individually, indicating that none of the F. tularensis bS21 homologs is individually essential. Our identification of *rpsU2* as an essential gene was likely due to the polar effects of transposon insertion into the first gene of an operon containing other known essential genes (*dnaG*, encoding primase, and *rpoD*, encoding the σ^70^ subunit of RNA polymerase). It is unclear if F. tularensis cells lacking all three *rpsU* genes are viable.

The literature reflects that bS21 may regulate gene expression in other bacteria. A recent study of the Flavobacterium johnsoniae ribosome revealed that bS21 plays a role in sequestering the anti-Shine-Dalgarno sequence (43). This occlusion occurs through contacts with the C-terminal region of bS21 that are conserved across Bacteroidetes species and provides a rationale to explain why most Bacteroidetes mRNAs lack Shine-Dalgarno sequences. Notably, the mRNA encoding bS21 in *F. johnsoniae* encodes a perfect Shine-Dalgarno sequence, strongly suggesting that bS21 regulates its expression through translational autoregulation (43). F. tularensis, however, is a member of the Gammaproteobacteria, has bS21 homologs that exhibit significant differences from F. johnsoniae at the C-terminal region, and encodes mRNAs that commonly contain sequences similar to the consensus Shine-Dalgarno sequence. This suggests that in F. tularensis, bS21 exerts its effects on gene expression differently.

In other bacteria that encode it, loss of bS21 leads to a variety of phenotypic changes. In Bacillus subtilis, loss of bS21 results in biofilm and motility defects (44), and in Listeria monocytogenes, inactivation of bS21 is linked to stress resistance and altered transcript abundance (45, 46). Staphylococcus aureus lacking functional bS21 exhibits increased resistance to the antibiotics daptomycin and vancomycin (47–49). Both Burkholderia pseudomallei and F. tularensis encode multiple bS21 homologs and in both organisms, virulence screens using transposon mutagenesis have identified one homolog as important for virulence (50, 51). Together, these findings suggest that bS21 may regulate gene expression in diverse bacterial species.

The idea that bS21 might modulate translation for a subset of mRNAs is further supported by the recent discovery that bS21 is encoded by thousands of sequenced bacteriophage genomes and is one of the most commonly encoded phage ribosomal proteins (52, 53). Transcripts encoding bS21 have been detected in metatranscriptomic samples along with transcripts for late-stage replication proteins (54) and at least one phage-encoded bS21 can be incorporated into E. coli ribosomes (52). All of this raises the possibility that the incorporation of a viral bS21 into the host ribosome may co-opt the translation machinery in favor of viral proteins and replication.

Our work, together with these earlier findings, strongly suggests that the incorporation of bS21 into the ribosome can impact the translation of a subset of mRNAs. Considering that bS21 can easily be exchanged among ribosomes, this provides an excellent mechanism to quickly fine-tune the cellular proteome. While the molecular mechanism leading to the modulation of translation has yet to be identified, it is reasonable to speculate that bS21 impacts translation during initiation through specific interactions with the 5′ untranslated regions of a specific set of mRNAs. These findings also support the idea that changes in ribosome composition may impact translation and provide another source for bacterial control of gene expression.

## MATERIALS AND METHODS

### Bacterial strains and growth conditions.

Unless otherwise noted, bacterial strains were grown as indicated here. Francisella tularensis subsp. *holarctica* live vaccine strain (LVS) cells were grown in Mueller-Hinton broth (BD Difco) supplemented with 0.025% iron pyrophosphate, 0.1% glucose, and 2% Isovitalex (sMHB), shaking aerobically or on cystine heart agar plates with 1% hemoglobin (CHA-H) at 37°C. Escherichia coli XL1-Blue cells were grown in lysogeny broth (LB) shaking aerobically or on LB agar plates at 37°C. Kanamycin was used at concentrations of 5 μg/mL (F. tularensis) or 50 μg/mL (E. coli).

### Vector construction.

Complementation plasmids for each bS21 homolog were created from a pKL42 (pF-PmrA-V), plasmid derived from pFNLTP6 (55). Specifically, the complementation plasmids produce bS21 homologs with a C-terminal VSV-G epitope under the control of the F. tularensis *groES* promoter. Each *rpsU* gene was amplified using a 5′ primer specifying an EcoRI site and an ideal Shine-Dalgarno sequence (5′-AGGAGG-3′) located six nucleotides upstream from the translation start site. The 3′ primer did not include the native stop codon and included DNA specifying a NotI site. The fragment was cloned into EcoRI/NotI digested pKL42, such that the 3′ end of each *rpsU* was in frame with codons specifying three alanines followed by the VSV-G epitope. The resulting plasmids were pKR6 pF-bS21-1-V, pKR7 pF-bS21-2-V, and pKR8 pF-bS21-3-V. The control plasmid pF was the original pFNLTP6 plasmid (containing the *groES* promoter but not any *rpsU* genes nor the VSV-G epitope).

The plasmid pEX18kan was modified to generate in-frame deletions of each *rpsU* gene as previously described (14). Flanking regions of ~600 bp from both sides of each *rpsU* gene were amplified by PCR. Primers amplifying the DNA adjacent to each *rpsU* gene included the first three or last three codons of the open reading frame and DNA specifying a NotI site, which also encodes an alanine linker (5′-GCGGCCGCT-3′). The two fragments were cloned into BamHI/KpnI-digested pEX18kan for each *rpsU* gene, respectively, yielding pKL122 pEXΔ*rpsU1*, pKR11 pEXΔ*rpsU2*, and pKR12 pEXΔ*rpsU3*; these plasmids were used to construct deletions via the allelic exchange as described below.

### Strain construction.

Deletion strains were constructed by allelic exchange as previously (56). Briefly, competent cells were made by washing F. tularensis LVS cells in 10% sucrose and resuspending them in an equal volume of 10% sucrose to cells. At least 1 μg of allelic exchange plasmid was electroporated into 50 μL competent cells in 0.2 cm cuvettes with a 2.5 kV pulse. Cells were allowed to recover in 4 to 5 mL sMHB for 4 to 8 h at 37°C, shaking. Cells in which a single integration event occurred were selected on CHA-H plates with kanamycin. These cells were subsequently plated on CHA-H containing 10% sucrose and lacking NaCl, allowing for survival only of cells that had crossed out the nonhomologous portion of the vector, including *sacB* and the kanamycin resistance gene. Colonies that were sucrose-resistant and kanamycin-sensitive were screened for deletions using PCR. Candidate strains were confirmed by amplification of genomic DNA outside the flanking regions on each side of the deletion and Sanger sequencing (Rhode Island Genomics and Sequencing Center). Plasmid pKL122 pEXΔ*rpsU1* was used to make LVS Δ*rpsU1*, plasmid pRK11 pEXΔ*rpsU2* was used to make LVS Δ*rpsU2*, and plasmid pKR12 pEXΔ*rpsU3* was used to make LVS Δ*rpsU3*.

Complementation plasmids were electroporated into LVS or LVS Δ*rpsU2* cells as described above and selected for on CHA-H plates with kanamycin.

### Immunoblotting.

Cells were collected from mid-log phase cultures (optical density at 600 nm (OD_600_) = 0.3 to 0.4) and resuspended in sample loading buffer (SLB: 1× NuPAGE LDS with 50 mM DTT) normalized to OD_600_ and heated at 95°C for 10 min. Cell lysates and fractions were separated by SDS-PAGE on 4 to 12% Bis-Tris NuPAGE gels in MES or MOPS running buffer (Invitrogen) and transferred to PVDF with the Mini Blot Module transfer system (Invitrogen; 20 V for 1 h on ice) or the Criterion cell for midi gels (Bio-Rad; 60 V for 40 min on ice) with 1× NuPAGE transfer buffer and 10% methanol. Whole-cell lysates were analyzed for total protein with the Invitrogen No-Stain Protein labeling reagent and all membranes were blocked with Odyssey blocking buffer diluted 1:5 in PBS overnight. For each antibody, the linear range of protein detection was determined by plotting sequential dilutions of one lysate from each strain as a standard curve to establish an appropriate volume of lysate to load. Membranes were probed with indicated monoclonal antibodies (BEI Resources, diluted 1:1000 in blocking buffer for all antibodies except anti-PdpB, which was diluted 1:250) or the VSV-G epitope (Sigma, diluted 1:2222). Proteins were detected using IRDye 800 CW donkey anti-mouse IgG or donkey anti-rabbit IgG (Li-Cor, diluted 1:10,000). Fluorescence was measured and quantified on the LiCor Odyssey CLx imager and software, and protein abundance was calculated relative to the total protein in each lane. Experiments were performed at least twice in biological triplicate and two to three technical replicates.

### RNA isolation and qRT-PCR.

Cells were collected from mid-log phase cultures (OD_600_ = 0.3 to 0.4). Nucleic acids were isolated using the Direct-Zol RNA purification kit (Zymo Research) according to the manufacturer’s protocol. Purified nucleic acids were treated with RQ1 DNase (Promega) for 1 h at 37°C and RNA was purified with the Direct-Zol RNA purification kit. cDNA was synthesized using Superscript III reverse transcriptase (Life Technologies) as previously described (14). qRT-PCR was performed using PowerUp SYBR Green Master Mix (Applied Biosystems) and a Roche LightCycler 480 (University of Rhode Island Genomics and Sequencing Center) essentially as described (14). Transcript abundances of *pdpA*, *pdpB*, *iglA*, and *pigR* were compared to three different control genes (*tul4*, *rpoA1*, and *bfr*) and since all results were similar, abundance is reported relative to *tul4*. Experiments comparing wild-type and *rpsU2* mutant cells were performed three times in biological triplicate. An experiment with cells lacking PigR was performed once.

### RNA-Seq.

Approximately 1.5 μg of RNA isolated as above was sent to the Microbial Genome Sequencing Center (MiGS) for RNA-Seq analysis, in biological triplicate (LVS pF) or duplicate (LVS Δ*rpsU2* pF, LVS Δ*rpsU2* pF-bS21-2-V). After using RiboZero Plus rRNA depletion, libraries were made using Illumina Stranded RNA library preparation and sequenced for a minimum of 12 million paired-end reads. Paired-end sequencing reads were mapped to the F. tularensis LVS genome (NCBI RefSeq accession number NC_007880) using bowtie2 version 2.2.4. Reads that mapped to annotated genes were counted using HTSeq version 0.11.2, and analysis of differential gene expression was conducted using DESeq2 version 1.32.0. Reported genes had a 2-fold higher or lower abundance than the wild type, all with an adjusted *P* value of 0.05 or lower.

### 70S ribosome purification.

70S ribosomes were isolated using sucrose cushion centrifugation essentially as described (57). Briefly, wild-type F. tularensis cells were grown in 500 mL sMHB to mid-log phase (OD_600_ = 0.3 to 0.4). Cells were chilled on ice for 20 min, centrifuged at 11,000 × *g* for 5 min at 4°C, then washed once with buffer H^10^M^10^A^1000^ (10 mM HEPES KOH pH 7.6, 10 mM MgCl_2_, and 100 mM NH_4_Cl) to remove ribonucleases. The pellet was then washed twice with buffer H^10^M^10^A^50^ (10 mM HEPES KOH pH 7.6, 10 mM MgCl_2_, and 50 mM NH_4_Cl, with or without 5 mM β-mercaptoethanol [BME]), and resuspended in ~15 mL of H^10^M^10^A^50^ with 20 U DNase I. Cells were lysed by passing through a French press three times at 800 lb/in^2^ and cell debris were removed by centrifugation at 146,000 × *g* for 15 min at 4°C. Supernatant was incubated with 0.5% Brij58 for 30 min and layered on top of H^10^M^10^A^500^ + 20% sucrose (10 mM HEPES KOH pH 7.6, 10 mM MgCl_2_, 500 mM NH_4_Cl, 20% sucrose, with or without 5 mM BME). Ribosomes were pelleted by ultracentrifugation in a 70 Ti rotor for 4 h at 146,000 × *g* at 4°C. The pellet was washed twice with H^10^M^10^A^50^ and gently resuspended in H^10^M^10^A^50^. This suspension was then layered onto another sucrose cushion (H^10^M^10^A^50^ with 40% sucrose) and centrifuged for 14 h at 146,000 × *g* at 4°C to further purify the ribosomes. Purified 70S ribosomes were gently resuspended in ~250 μL of H^10^M^10^A^50^ and stored at −80°C.

### LC-MS/MS of purified LVS ribosomes.

70S ribosomes from wild-type LVS cells were prepared as described above. Samples were either purified via gel stacking before mass spectrometry analysis or maintained in H^10^M^10^A^50^ and delivered to the Northwestern Proteomics Core. The proteins were in-gel digested or in-solution digested and liquid chromatography-tandem mass spectrometry (LC-MS/MS) analysis was completed based on internal protocols, matching peptides to the F. tularensis LVS proteome (NC_007880).

### DIA mass spectrometry.

Cells were collected from mid-log phase cultures (OD_600_ = 0.3 to 0.4) and resuspended in Buffer 1 (20 mM HEPES KOH pH 7.9, 50 mM KCl, 0.5 mM DTT) with protease inhibitor tablets (Complete Mini, EDTA-free, Roche). Cells were lysed by sonication and protein concentration was determined using a BCA protein assay (Pierce). Lysates with concentrations between 620 and 862 μg/mL were used by the University of Arkansas for Medical Sciences (UAMS) Proteomics Core for analysis. Protein extraction and protease digestion were completed according to UAMS internal protocols. Data-independent acquisition (DIA) was completed with the Orbitrap Exploris 480 mass spectrometer.

### Polysome purification and sucrose gradient sedimentation.

Polysomes were isolated essentially as described (58). F. tularensis cells were grown until early log (OD_600_ 0.2 to 0.25). Liquid cultures were rapidly filtered through 0.2 μm nitrocellulose membranes and transferred to a conical tube filled with liquid nitrogen. Cells were lysed by bead-beating with 650 μL flash frozen lysis buffer (25 mM HEPES KOH pH 7.6, 100 mM NH_4_Cl, 10 mM MgCl_2_, 0.4% Triton X-100, 0.1% NP-40, 100 U/mL RNase-free DNase) using the TissueLyser II (Qiagen) five times (15 Hz, 3 min). Cell debris was pelleted and the polysome-containing lysates were stored at −80°C.

Sucrose gradients were prepared using 10% and 55% sucrose solutions in 25 mM HEPES pH 7.6, 100 mM NH_4_Cl, 10 mM MgCl_2_ with the BioComp Instruments 153 Gradient Station (BioComp). Cell lysates were layered onto gradients and centrifuged with the Beckman-Coulter SW40 Ti rotor at 40,000 rpm for 2.5 h at 4°C. Gradients were fractionated using the Triax full spectrum flow cell and fractionator (BioComp; 0.2 mm/s, 28 fractions), and A260 was measured every second. Collected fractions were stored at −80°C. 20 μL of each fraction was combined with 10 μL of sample loading buffer (3× NuPAGE LDS with 50 mM DTT) and immunoblotted as described above.

### Intramacrophage replication assays.

Intramacrophage growth assays were performed as previously described (56). Briefly, approximately 2.5 × 10^4^ cells of murine macrophage-like J774A.1 cells were incubated at 37°C in 5% CO_2_ overnight in 96-well plates in DMEM (Invitrogen) supplemented with 10% fetal bovine serum (Gemini Bio-Products; DMEM-F). Macrophage cells were infected with LVS and indicated derivative strains at an MOI of approximately 5 to 10. After 2 h, cells were washed twice with PBS, and the medium was replaced with DMEM-F containing 10 μg/mL gentamicin. After 2 h or 24 h of infection, macrophages were lysed for 30 min in 1% saponin in PBS and plated for enumeration.

### Antibiotic chase experiment.

Indicated F. tularensis LVS cells were grown to mid-log phase in liquid culture (OD_600_ = 0.3 to 0.4). Spectinomycin was added to a final concentration of 200 μg/mL. Cells were collected at the indicated time points after antibiotic addition and resuspended in sample loading buffer normalized to OD_600_ at *t* = 0. Immunoblotting was conducted as described above and analysis was conducted using a one-phase decay equation on Prism 9 (GraphPad). Data represent two experiments in biological triplicate.

### Data availability.

RNA-Seq reads are available in the National Center for Biotechnology Information Gene Expression Omnibus (NCBI GEO) under accession number GSE210766.
