# Supplementary material for: A Ribosomal Protein Homolog Governs Gene Expression and Virulence in a Bacterial Pathogen
Source: J Bacteriol. 2022 Sep 19;204(10):e00268-22. doi: 10.1128/jb.00268-22 (PMC9578407; doi:10.1128/jb.00268-22)
Supplement: Supplemental file 1 — Fig. S1 to S5. Download jb.00268-22-s0001.pdf, PDF file, 4.9 MB [file jb.00268-22-s0001.pdf]

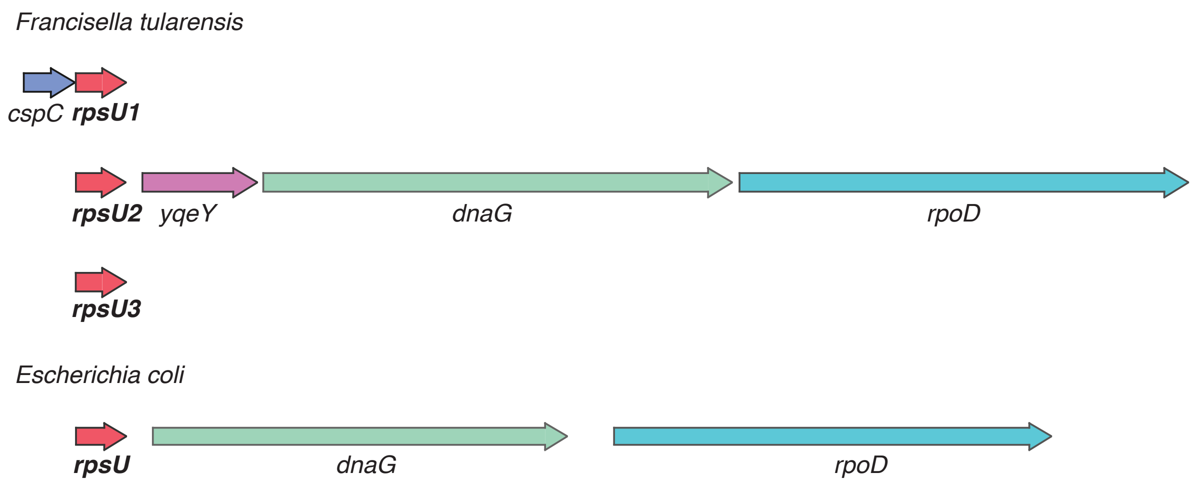

Figure S1. *F. tularensis* encodes three *rpsU* genes. *F. tularensis rpsU2*, which encodes bS21-2, is syntenic with the only *rpsU* in *E. coli*, which is located in the macromolecular synthesis operon (1). This operon in *E. coli* includes *rpsU* (encoding bS21), *dnaG* (encoding DNA primase), and *rpoD* (encoding RNA polymerase σ^{70}). In *F. tularensis*, this operon also includes *yqeY*, the product of which may be involved in tRNA aminoacylation. *rpsU1*, encoding bS21-1, is located immediately downstream of *cspC* (encoding cold-shock protein CspC), while *rpsU3*, encoding bS21-3, is not apparently in an operon with other genes. Genomic locations of *rpsU* genes were determined using RefSeq NC_007880 for *F. tularensis* and NC_000913 for *E. coli*.

	F. tularensis subsp *holarctica* LVS bS21-1	*F. tularensis* subsp *holarctica* LVS bS21-3	*F. tularensis* subsp *holarctica* LVS bS21-2	*E. coli* bS21
F. tularensis subsp *holarctica* LVS bS21-1	100.0	72.3	54.0	50.8
F. tularensis subsp *holarctica* LVS bS21-3		100.0	47.6	48.5
F. tularensis subsp *holarctica* LVS bS21-2			100.0	60.0
E. coli bS21				100.0

Figure S2. The three bS21 homologs in *F. tularensis* are distinct. Percent identities of amino acid sequences for *F. tularensis* LVS bS21-1, bS21-2, bS21-3, and *E. coli* bS21 were calculated using the multiple sequence alignment tool ClustalOmega (2). The bS21 homologs in *F. tularensis* are similar to each other, particularly bS21-1 and bS21-3 which are 72% identical at the amino acid level. bS21-2, encoded by the *rpsU* homolog gene syntenic to the single *E. coli rpsU* gene, is also the most similar to *E. coli* bS21, with 60% amino acid identity.

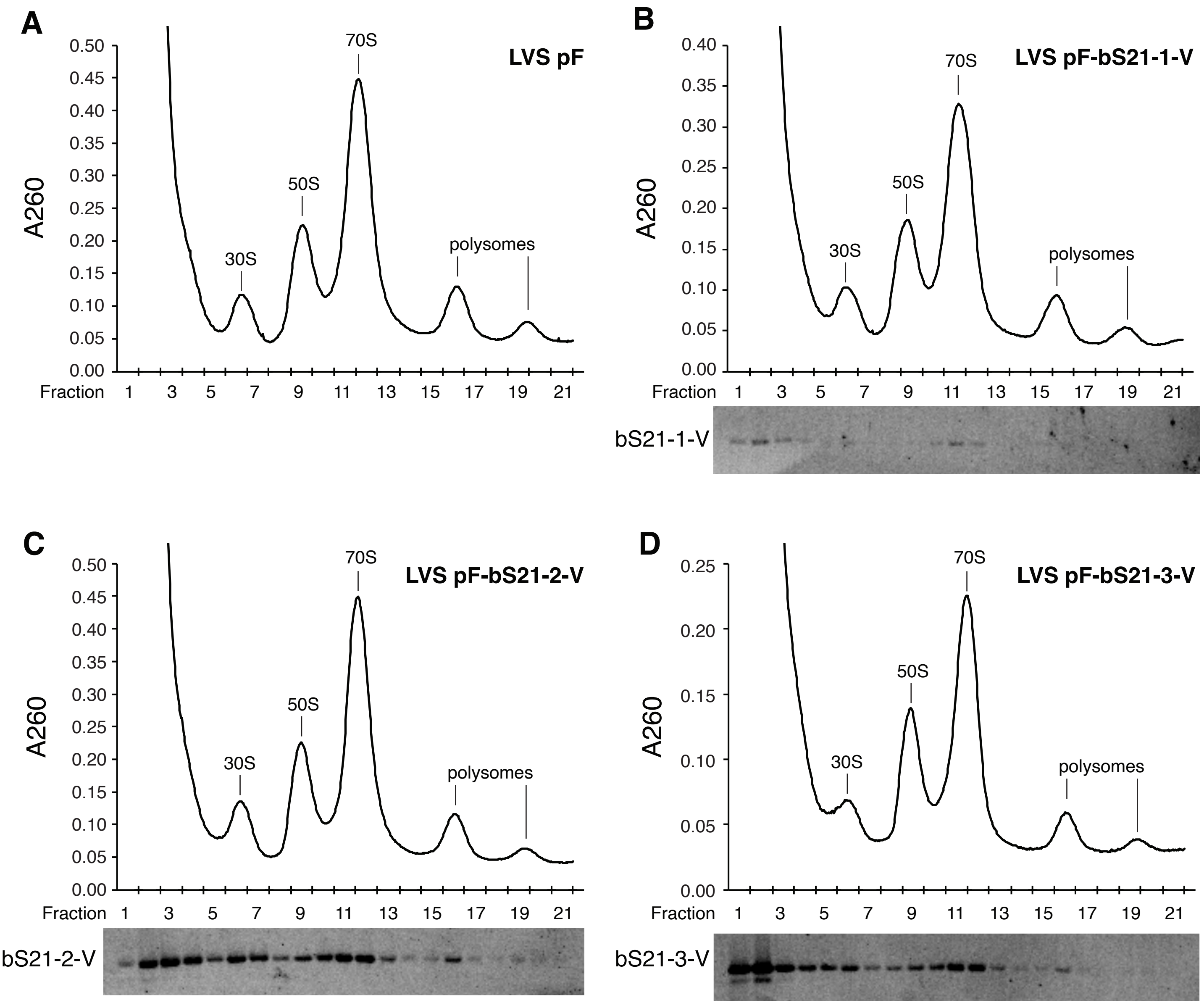

Figure S3. Each bS21 homolog can be detected in translationally-active ribosomes. For **A – D**, top: Sucrose gradient sedimentation profile from actively-translating wild-type *F. tularensis* cells with either empty vector or ectopic expression of indicated bS21 homolog. Nucleic acid content was monitored by A260 (y-axis). Peaks corresponding to the 30S, 50S, 70S, and polysomes are indicated. Fractions collected are indicated on the x-axis. For **A – D**, bottom: Immunoblot analysis of fractions from sucrose gradient sedimentation (above), probing for VSV-G. Wells correspond to fractions 1 – 21 from profile above. **A.** Cells from wild-type *F. tularensis* LVS with empty vector (LVS pF). **B.** Cells from wild-type *F. tularensis* LVS with ectopic expression of bS21-1 (LVS pF-bS21-1-V). **C.** Cells from wild-type *F. tularensis* LVS with ectopic expression of bS21-2 (LVS pF-bS21-2-V). **D.** Cells from wild-type *F. tularensis* LVS with ectopic expression of bS21-3 (LVS pF-bS21-3-V).

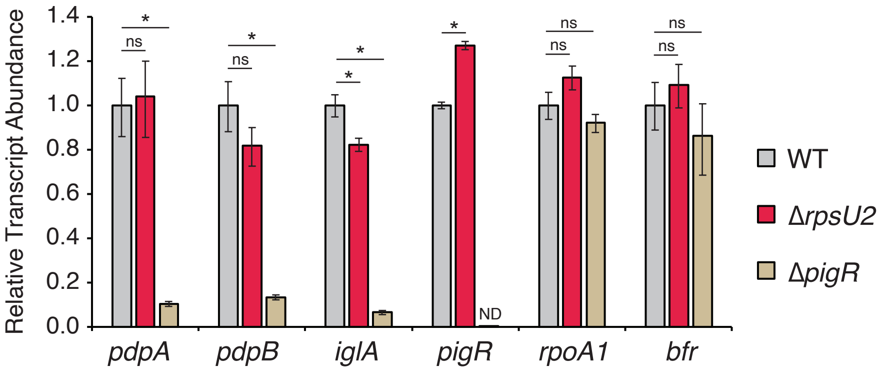

Figure S4. Loss of bS21-2 does not affect transcript abundance of FPI-encoded genes. Quantitative real-time PCR was used to determine the relative transcript abundance for indicated FPI genes in wild-type cells, cells lacking bS21-2 (Δ*rpsU2*), or cells lacking the transcription factor PigR (Δ*pigR*). Cells lacking PigR serve as a positive control, as PigR positively regulates its own transcription and the transcription of *pdpA*, *pdpB*, and *iglA*. The *rpoA1* and *bfr* genes are included as negative controls, as their expression is not influenced by bS21-2 or PigR. Transcript abundances are normalized to *tul4*, whose expression is not influenced by bS21-2 or PigR. Error bars represent 1 SD from the value (calculated using the mean threshold cycle). ns: not significant. ND: not detected. *adjusted p < 0.05 by t-test.

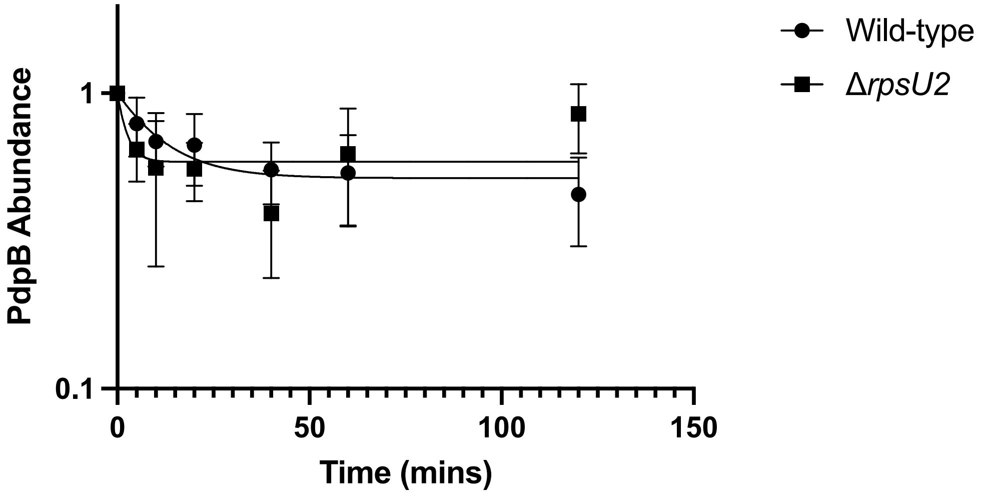

Figure S5. Loss of bS21-2 does not affect protein degradation of PdpB. One-phase decay of PdpB from antibiotic-chase experiment from wild-type cells and cells lacking bS21-2 (Δ*rpsU2*). Neither strain showed significant degradation of PdpB through the time points assessed; the calculated half-life for both was greater than 120 minutes. Y-axis is logarithmic and error bars represent 1 SD from the mean.

Supplemental References

1. Lupski JR, Godson GN. 1984. The *rpsU-dnaG-rpoD* macromolecular synthesis operon of *E. coli*. Cell 39:251–252.

2. Madeira F, Pearce M, Tivey ARN, Basutkar P, Lee J, Edbali O, Madhusoodanan N, Kolesnikov A, Lopez R. 2022. Search and sequence analysis tools services from EMBL-EBI in 2022. Nucleic Acids Res 50:W276–W279.
